# Biophysical Characterization of the Strong Stabilization of the RNA Triplex poly(U)_•_poly(A)_*_poly(U) by 9-O-(ω-amino) Alkyl Ether Berberine Analogs

**DOI:** 10.1371/journal.pone.0037939

**Published:** 2012-05-29

**Authors:** Debipreeta Bhowmik, Suman Das, Maidul Hossain, Lucy Haq, Gopinatha Suresh Kumar

**Affiliations:** 1 Biophysical Chemistry Laboratory, Chemistry Division, CSIR-Indian Institute of Chemical Biology, Kolkata, India; 2 Department of Chemistry, Jadavpur University, Kolkata, India; Institute of Molecular and Cell Biology, Singapore

## Abstract

**Background:**

Binding of two 9-O-(ω-amino) alkyl ether berberine analogs BC1 and BC2 to the RNA triplex poly(U)_•_poly(A)_*_poly(U) was studied by various biophysical techniques.

**Methodology/Principal Findings:**

Berberine analogs bind to the RNA triplex non-cooperatively. The affinity of binding was remarkably high by about 5 and 15 times, respectively, for BC1 and BC2 compared to berberine. The site size for the binding was around 4.3 for all. Based on ferrocyanide quenching, fluorescence polarization, quantum yield values and viscosity results a strong intercalative binding of BC1 and BC2 to the RNA triplex has been demonstrated. BC1 and BC2 stabilized the Hoogsteen base paired third strand by about 18.1 and 20.5°C compared to a 17.5°C stabilization by berberine. The binding was entropy driven compared to the enthalpy driven binding of berbeine, most likely due to additional contacts within the grooves of the triplex and disruption of the water structure by the alkyl side chain.

**Conclusions/Significance:**

Remarkably higher binding affinity and stabilization effect of the RNA triplex by the amino alkyl berberine analogs was achieved compared to berberine. The length of the alkyl side chain influence in the triplex stabilization phenomena.

## Introduction

Triple helix formation has been proposed as a means for sequence-specific recognition and binding by a relatively short single stranded DNA or RNA to either a double-stranded DNA or RNA [1]–[7] originally discovered by Rich and coworkers as early as in 1957 [8]. The binding of the third strand on the duplex to form the triplex occurs essentially by Hoogsteen base paired H-bonding interactions in the major groove of the duplex. The antiparallel arrangement of a purine strand flanked by two pyrimidine strands is termed as the ‘pyrimidine-purine_*_pyrimidine’ (YRY, Y is pyrimidine and R is purine) motif that yields C.G_*_C^+^, T.A_*_T (DNA triplexes) or the U.A_*_U triplex (RNA triplex) (symbols . and _*_ represent Watson-Crick and Hoogsteen base pairing strands of the triplex). Triplex nucleic acids may have functional roles in many cellular processes such as transcriptional regulation, post-transcriptional RNA processing, modification of chromatin structure, DNA repair etc. For example, DNA triplex formation may be involved the *c-myc* proto-oncogene regulation [9] and RNA triplexes may exist within transfer RNA [10], [11] the *Tetrahymena* group I intron [12]–[14], the human immunodeficiency virus TAR RNA [15]–[17] HIV frame shift signal etc [18]. The discovery of the triplex-unwinding helicases [19] as well as the recent revelation of the genomics and many critical biological roles of the non-coding RNAs [20] necessitate the speculation of the involvement of triple-helices in many critical roles in the cell [21]–[24]. The potential in vivo roles and importance of nucleic acid triple helical structures have been reviewed in great details recently [25].

Due to Hoogsteen base pairing, stability of the triplex structure is relatively lower compared to the corresponding duplex and the third strand dissociates at a much lower temperature compared to the duplex denaturation temperature. This is a critical limitation in their utility and application in vivo as probes of structure, inhibitors of protein synthesis as well as therapeutic agents. Therefore, molecules having the ability to recognize, bind and stabilize specific sequences of triple helical nucleic acid structures are of particular interest in antigene therapy. Recently several biophysical studies on the properties of DNA triple helices [2], [3], [26], [27] and many small molecules that can enhance the stability of triplexes have been described [28]–[31]. But most of these are limited to DNA triplexes and studies on the stabilization of RNA triplexes and correlating thermodynamic factors to the structural aspects that actually account for the observed differences in stability are scarce. Our laboratory has shown that many isoquinoline alkaloids can bind and significantly stabilize RNA triplexes [32].

Berberine is a natural alkaloid with remarkable biological relevance and utility, and represents a potential molecule for drug screening and development. The antiproliferative property and sensitivity enhancement of berberine to various cancer cell lines [33]–[39] have led to significant interest in this molecule as a prospective anticancer drug candidate [40], [41]. The potential biotargets of berberine may include DNA topoisomerases, telomerase, and DNA or RNA structures. In particular, DNA and RNA have long been thought to be the target in manifesting its anticancer activities, and accordingly, the binding properties to single strands, duplex, triplex, and quadruplex structures have been described in many recent publications and reviews [42]–[44]. Notably, these studies have revealed an unusually high affinity and induction of self-structure formation by berberine to poly(A) [45], [46]. Currently, RNA molecules are thought to be involved in many cellular activities and RNA triplex structures may be a potential structural motif for therapeutic intervention.

**Figure 1:**
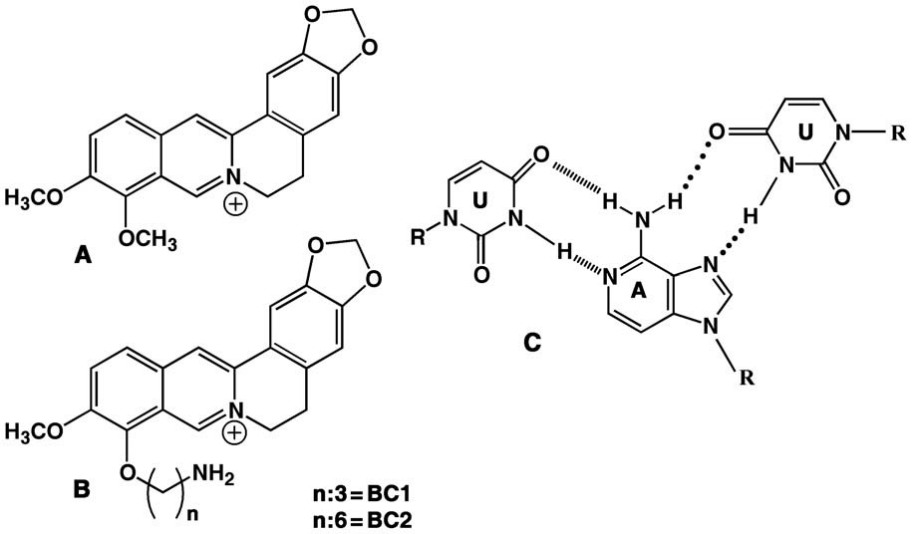
Chemical structures of BC, analogs and the base triplet of poly(U)_•_poly(A)_*_poly(U). (A) BC (B) BC analogs and (C) triplex.

In order to improve the efficacy of berberine and make it a viable therapeutic agent many synthetic strategies have been adopted recently [47]–[50]. Substitution on the isoquinoline moiety has been an important approach and the enhanced effect of 9-substituted analogs on drug-topoisomerase II interactions is well documented several years ago [51]. The 9-substituent within the domain has a major influence, presumably by facilitating drug interaction with enzyme and/or enzyme-DNA complexes and many were synthesized to enhance the DNA binding as well as pharmacological activities [52]–[56]. In this manuscript we describe the triplex-binding affinities and the associated thermodynamic aspects of the interaction of two new 9-amino alkyl analogs (Figure 1) in comparison to berberine. These functional substituents depending on the number of CH_2_ group have been documented to increase duplex DNA binding affinities and also induce self-structure formation in poly(A) [46], [57]. Thus, the interaction studies on these berberine derivatives with structural alteration at the 9-position may provide key insights into the different aspects of association process of berberine with RNA. In the light of remarkably strong affinity of these molecules to various nucleic acid structures as mentioned above in this study we investigated the structural aspects in conjunction with energetics of the interaction of two representative analogs with the RNA triplex poly(U)_•_poly(A)_*_poly(U) (RNA triplex hereafter).

## Materials and Methods

### Biochemicals

Polynucleotide samples of double stranded poly(A)_ •_poly(U) and single stranded poly(U) were obtained from Sigma-Aldrich Corporation (St. Louis, MO, USA) and were used as received. Berberine was obtained from Aldrich and its analogs were prepared and characterized as described in [58].

### Preparation of Stock Solutions

The RNA triplex was prepared as reported earlier [32]. Concentration was determined using molar extinction coefficients (ε) reported in the literature [59]. For this study two 9-amino alkyl berberine analogs (Figure 1) with chain length of 3 and 6 CH_2_ groups were used. They were fairly soluble in aqueous buffers, and hence their solutions were freshly prepared each day and kept protected in the dark until use. The concentration of berberine was determined by an ε value of 22,500 M^−1^ cm^−1^ at 345 nm and that of the 9-analogs using a common ε value of 22,500 M^−1^ cm^−1^ at 345 nm. No deviation from Beer’s law was observed for the alkaloids in the concentration range employed in this study. All experiments were performed in 10 mM sodium cacodylate buffer containing 25 mM NaCl, 0.1 mM Na_2_EDTA, pH 7.0 (total [Na^+^]  = 35 mM). Glass distilled deionized water and analytical grade reagents were used for the preparation of the buffer. All buffer solutions were filtered through Millipore filters (Millipore India Pvt. Ltd., Bangalore, India) of 0.22 µm pore size before use. The triplex structure was characterized by circular dichroic spectral pattern and biphasic optical melting profile [59]–[60].

### Absorbance Spectroscopy

Spectrophotometric titration of alkaloid analogs with the triplex was performed on a Jasco V660 double beam double monochromator spectrophotometer (Jasco, Hachioji, Japan) at 25±0.5°C using the methodologies described previously [61], [62]. Matched quartz cuvettes of 1 cm path length were used. A typical titration was carried out by keeping a fixed known concentration of the triplex solution in the sample and reference cuvettes and adding successively small aliquots of a known concentrated stock of the alkaloid into the sample cuvette and equivalent amount of buffer into the reference cuvette. After each addition, the system was allowed to equilibrate for at least 5 min. before noting the absorbance at the wavelength maximum (A_max_) of the alkaloid and the isosbestic point (A_iso_). The data obtained from these titrations were quantified by constructing Scatchard plots [63].

### Fluorescence Spectroscopy

Fluorescence titrations were performed on either a Shimadzu RFPC fluorimeter (Shimadzu Corporation, Kyoto, Japan) or Hitachi F4010 unit (Hitachi, Tolyo, Japan) in fluorescence free quartz cuvettes of 1 cm path length in sample holder thermo regulated at 25±0.5°C using an Eyela Uni Cool U55 water bath (Tokyo Rikakikai Co. Ltd. Tokyo, Japan). The uncorrected emission spectra of the alkaloid and analogs were at first recorded and then titrated with aliquots of triplex solution of high concentration to prevent dilution effects. The samples were excited at 350 nm and all the measurements were performed under conditions of stirring.

### Evaluation of Binding Affinity from Spectroscopic Results

In absorption spectroscopy, following each addition of the alkaloid to the RNA triplex solution (50 µM), the total alkaloid concentration (C_t_) present was calculated as 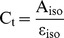 from the absorbance at the respective isosbestic point (A_iso_) where ε_iso_ is the molar extinction coefficient at the isosbestic point. The expected absorbance (A_exp_) at the wavelength maximum was calculated using the relation 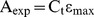 where ε_max_ is the molar extinction coefficient at the wavelength maximum. The difference in A_exp_ and the observed absorbance (A_obsd_) was then used to calculate the amount of bound alkaloid as 
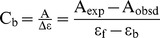
. The concentration of the free alkaloid was determined by the difference, 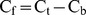. The extinction coefficient of the completely bound alkaloid was determined by mixing a large excess of the RNA triplex to a known quantities of the alkaloids assuming total binding, 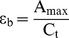. From the fluorescence data, C_b_ was calculated using the relation 
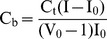
 **_,_** where C_t_ is the total alkaloid concentration, I is the observed fluorescence intensity, I_o_ is the fluorescence intensity of identical concentration of the alkaloid in the absence of the triplex, and V_o_ is the experimentally determined ratio of the fluorescence intensity of totally bound alkaloid to that of the free alkaloid. Free alkaloid concentrations (C_f_) were obtained from the relationship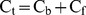. The binding ratio r is defined as 
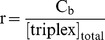
.

These data were cast into Scatchard plots of r/C_f_ versus r. The binding isotherms with negative slopes at low r values were analyzed according to the excluded site model for a non-linear non-cooperative ligand binding system using the following equation
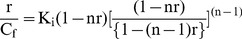
(1)where *K*_i_ is the intrinsic binding constant to an isolated binding site, and n is the number of nucleotides excluded by the binding of a single alkaloid molecule. All the binding data were analyzed using Origin 7.0 software (Microcal Inc., Northampton, MA, USA) to determine the best-fit parameters of *K*_i_ and n as described in details earlier [64].

### Optical Thermal Melting Experiments

Optical thermal melting of the triplex and triplex-alkaloid complexes was monitored by UV absorption at 260 nm and was performed using a Shimadzu Pharmaspec 1700 unit equipped with a peltier controlled TMSPC–model accessory (Shimadzu Corporation, Kyoto, Japan) monitoring the absorbance change at 260 nm as described earlier [65], [66]. The temperature of the sample was increased at a rate of 1.0°C /min, typically starting at 20°C and ending at 70°C when both transitions were completed.

### Measurement of Energy Transfer

Energy transfer from the triplex to the bound alkaloid was measured from the excitation spectra of the complex in the wavelength range 220–310 nm [67]–[69]. Excitation spectra were recorded at an emission wavelength of 520 nm for BC and analogs. The ratio 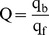, where q_b_ and q_f_ are the quantum efficiencies of bound and free alkaloid, respectively, was calculated for each wavelength using the equation 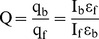 where I_b_ and I_f_ are the fluorescence intensities of the alkaloids in the presence and absence of the triplex, respectively, and ε_b_ and ε_f_ are the corresponding alkaloid molar extinction coefficients. A plot of the ratio, Q_λ_/Q_310_ against wavelength was constructed. The wavelength of 310 nm was chosen as the normalization wavelength due to the negligible absorbance for triplex at this wavelength.

### Fluorescence Polarization Anisotropy Measurements

Fluorescence polarization anisotropy measurements of alkaloids and triplex complexes were executed on a Hitachi F4010 fluorimeter (Hitachi Ltd. Tokyo, Japan) at 25^o^. Steady state polarization anisotropy ‘A’ is defined as
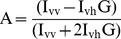
(2)where G is the ratio I_hv_/I_hh_ used for instrumental correction. I_vv_, I_vh_, I_hv_ and I_hh_ represent the fluorescence signal for excitation and emission with the polarizer positions set at (0°, 0°), (0°, 90°), (90°, 0°) and (90°, 90°), respectively.

### Fluorescence Quenching Experiments

Fluorescence quenching experiments were performed by mixing, in different ratios, two solutions, viz. KCl and K_4_[Fe(CN_6_], at a fixed total ionic strength. At a constant P/D (RNA triplex/alkaloid molar ratio) fluorescence intensity was monitored as a function of varying concentration of the ferrocyanide as described previously [32], [70], [71]. The data were plotted as Stern-Volmer plots of relative fluorescence intensity (F_o_/F) versus [Fe(CN_6_]^4−^ concentration according to the Stern-Volmer equation, 
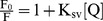
, where F_o_ and F denote the fluorescence emission intensities in the absence and presence of [Fe(CN_6_]^4−^ and [Q] is the quencher concentration. *K*_SV_ is the Stern-Volmer quenching constant, which gives an estimate of the efficiency of quenching by the quencher.

### Viscosity Measurements

A Cannon-Manning semi micro capillary viscometer (size 75, Canon Instruments Co. State College, PA, USA) kept thermostated at 25±1.0°C was used to measure the time required for flow to calculate the viscosity of the RNA triplex-alkaloid complexes. Relative viscosities for RNA triplex either in presence or absence of the alkaloids were calculated as reported earlier [72]. The relative increase in length, L/L_o_, may be obtained from the corresponding increase in relative viscosity with the use of the equation, 
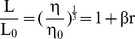
, where L and L_o_ are the contour lengths of triplex in presence and absence of the alkaloids, η and η_o_ are the corresponding values of intrinsic viscosity (approximated by the reduced viscosity 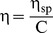, where C is the triplex concentration). β is the slope of a plot of L/L_o_ *versus* r where a slope of ∼2 may suggest true intercalative binding.

### Circular Dichroism Studies

Circular dichroism (CD) spectra were measured on a PC controlled spectropolarimeter, JASCO J815 unit (Jasco, Hachioji, Japan) equipped with a temperature programmer (model PFD 425L/15) at 25±0.5^o^. A rectangular strain free quartz cuvette of 1 cm path length was used. Each spectrum, averaged from four successive accumulations at a scan rate of 100 nm/min. keeping a bandwidth of 1.0 nm at a sensitivity of 100 milli degrees, was baseline corrected, smoothed and normalized to nucleotide phosphate concentration in the region 220–400 nm. The molar ellipticity (θ) values are expressed in deg. cm^2^ dmol^−1^.

### Isothermal Titration Calorimetry

Isothermal titration calorimetry (ITC) was performed on a MicroCal VP-ITC microcalorimeter (MicroCal, Inc., Northampton, MA, USA). Instrument control data acquisition, and analysis were carried out using the in built Origin software 7.0 package using standard protocols reported earlier [66], [70], [73]. All experiments were conducted at 25°C. Aliquots (10 µL) of the alkaloid solution were injected from the rotating syringe (286 r.p.m) into the isothermal sample chamber containing 1.4235 ml of the triplex solution (25 µM). Control experiments to estimate the heat of dilution of the alkaloids were performed by titrating the alkaloid solutions to the buffer kept in the calorimeter cell under the same experimental conditions and protocols. Heat of dilution of the triplex titration into buffer was found to be negligible. Analysis of the integrated heat data was performed using Origin 7.0.

Experimental data were fitted using a non-linear least-squares minimization algorithm to a theoretical titration curve using model of one site binding site Levenberg- Marquardt non-linear least squares curve fitting algorithm equations included in the software package of Origin 7.0 to estimate the binding affinity (*K_a_*), the binding stoichiometry (N) and the enthalpy of binding (Δ*H* ^o^). The binding Gibbs energy (Δ*G* ^o^) and the entropic contribution (TΔ*S* ^o^) to the binding were subsequently calculated from standard relationships described earlier [65], [73].

## Results and Discussion

### Electronic Absorption Spectral Studies of the Binding

**Figure 2:**
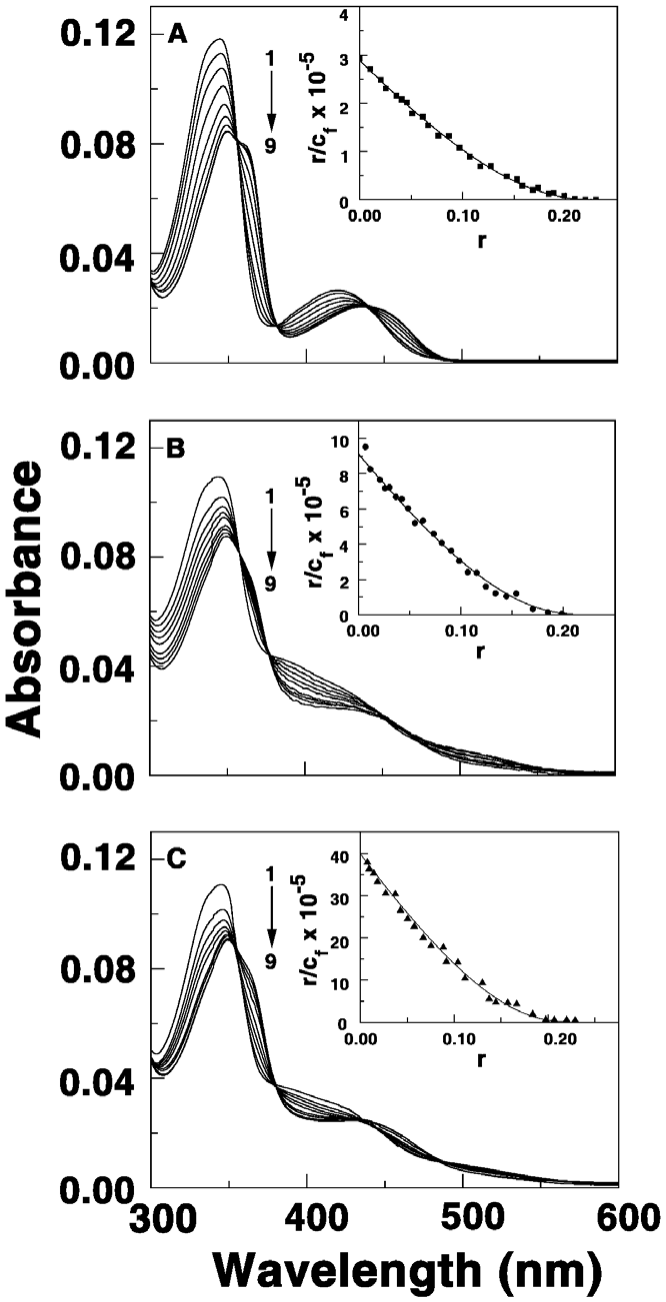
Absorption spectral change of alkaloids in presence of triplex. (A) BC (B) BC1 (C) BC2 each of 5.0 μM treated with increasing concentrations of poly(U)_•_poly(A)_*_poly(U). P/D saturation for BC, BC1 and BC2 were 28.0, 20.0 and 15.0, respectively. Inset: respective non-cooperative Scatchard isotherms of the binding.

The binding of the berberine analogs to the RNA triplex was at first investigated by absorption spectral measurements. Berberine and its analogs have characteristic visible absorption spectra with intense peaks around 345 and 420 nms (Curve 1 of Fig. 2). As aliquots of the triplex was added to the spectrophotometric cuvette containing berberine and analogs, the intensity of the peaks decreased with concomitant red shift of the peak maxima generating sharp isosbestic points. Typical absorption spectral changes for BC, BC1 and BC2 in presence of increasing concentrations of the triplex are presented in Figure 2. Hypochromic and bathochromic shifts of ligand centered transitions for aromatic ligands typically arise from *π*-stacking interactions which may result from intercalation with the base triplets. Berberine has been revealed to intercalate between DNA bases from the recent X-ray crystal structure data [43]. Similarly, berberine analogs (Figure 1) should be able to intercalate between the base triplets without large steric constraints and the alkyl side chain may provide additional interaction modules in the grooves or with the functional groups on the base triplets. The presence of clear isosbestic points suggest that the alkaloid analogs bind to triplex by equilibrium enabling the estimation of the binding affinity by Scatchard analysis in comparison to BC. A summary of the optical properties of free and poly(U).poly(A)*poly(U) bound alkaloid analogs are presented in Table S1.

### Spectrofluorimetric Studies

**Figure 3:**
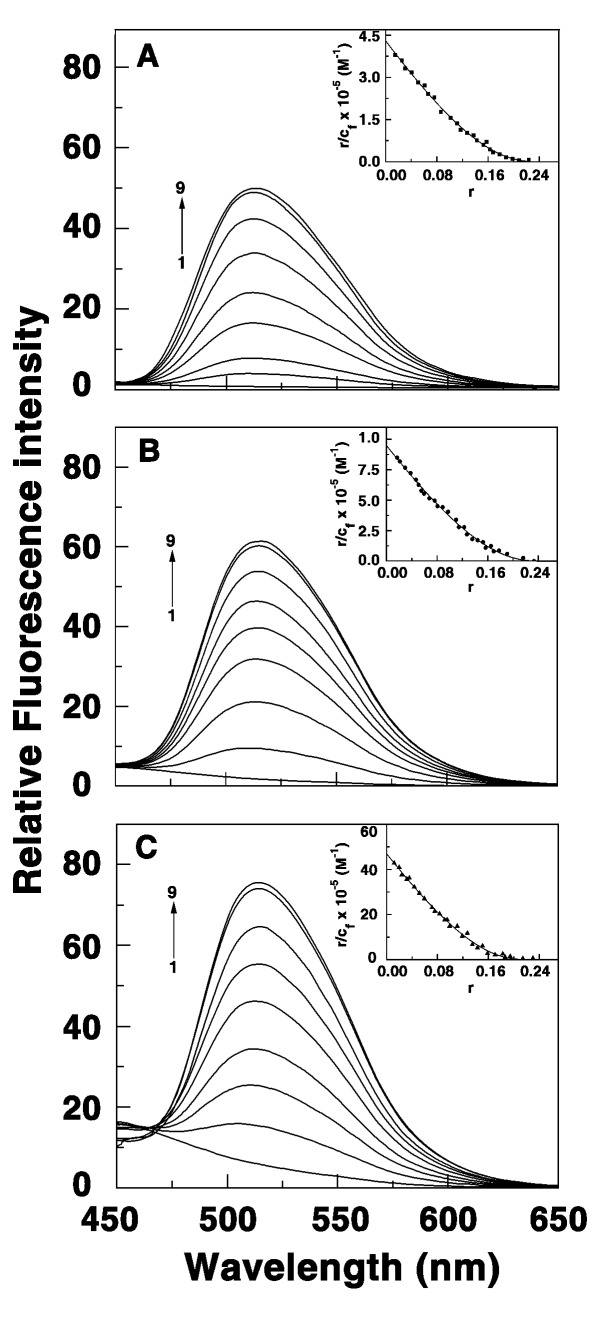
Fluorescence spectra of BC and BC analogs in presence of triplex. (A) BC (B) BC1 (C) BC2 (2.0 μM each) treated with increasing concentration of RNA triplex. P/D saturation for BC, BC1 and BC2 were 32.0, 24.0 and 18.0, respectively. Inset: respective non-cooperative Scatchard plots of binding.

Berberine and its 9-amino alkyl analogs are weak fluorophores with low fluorescence emission intensity that enhanced several-fold on binding to nucleic acids [46], [47]. In the presence of the triplex, the intrinsic fluorescence of the analogs registered remarkably large enhancement of the fluorescence intensity reaching a saturation that can be used to evaluate the binding affinities. The large enhancements may also be in support of the hydrophobic position of the bound molecules between the base triplets. Typical fluorescence spectral changes of BC, BC1 and BC2 in presence of the RNA triplex are presented in Figure 3.

### Evaluation of the Binding Affinities from Absorbance and Fluorescence Titration Data

The spectral changes in the absorption and fluorescence titration data were expressed as Scatchard plots and they are depicted in the inset of Fig. 2 and 3. The Scatchard plots had negative slopes at low r values enabling further analysis of the curved isotherms by the McGhee-von Hippel methodology [74] for non-cooperative binding for evaluation of the binding constants and the number of excluded sites on binding of a single alkaloid molecule. From this analysis, it was found that the binding affinity values (*K_i_*) of BC, BC1 and BC2 to the triplex were 2.88±0.02×10^5^ M^−1^, 8.65±0.34×10^5^ M^−1^, and 3.87±0.41×10^6^ M^−1^, respectively, from absorption spectra. The number of excluded sites was around 4.2, 4.2 and 4.3, respectively, for BC, BC1 and BC2. Similarly spectrofluorimetric analysis of the binding resulted in values with magnitudes close to that obtained from absorption spectroscopy and these are presented in Table 1. It can be seen that the binding affinity BC1 and BC2 to the RNA triplex was higher by 3 and 15 times than that of BC suggesting that the alkyl chain remarkably influence the triplex binding.

**Table 1:** Binding parameters for poly(U)•poly(A)_*_poly(U) triplex-alkaloid complexation obtained from McGhee-von Hippel analysis of the Scatchard plot from the spectrophotometric and fluorimetric titration data at 25°C^a^.

Alkaloid	Absorbance		Fluorescence	
	*K_i_ */10^5^ M^−1^	n	*K_i_ */10^5 ^M^−1^	n
BC	2.88±0.02	4.3	3.9±0.05	4.1
BC1	8.65±0.34	4.2	9.5±0.10	4.0
BC2	39.87±0.41	4.3	47.0±0.56	4.3

a Average of four determinations in each case. *K_i_* is the intrinsic binding constant to an isolated binding site and ‘n’ represents the number of excluded sites.

### Complex Formation Stoichiometry (Job plot)

The binding stoichiometry of association of these analogs with the RNA triplex and the possible number of binding sites were determined independently by continuous variation analysis (Job plot) [75], [76] in fluorescence. In Job plot, the ligand: RNA molar ratio was varied while the total molar concentration remains constant. The stoichiometry of binding is determined by the molar ratio where maximal binding is observed. The plot of difference fluorescence intensity (ΔF) at respective λ_max_, for BC, BC1 and BC2, versus their mole fractions presented in Figure S1, revealed a single binding mode for both these molecules on the RNA-triplex. From the inflection points χ = 0.190, 0.186 and 0.178 (indicated by arrow), the number of nucleotides bound per BC, BC1and BC2 can be estimated to be around 4.26, 4.37 and 4.6, respectively. This is in good agreement with the number of binding sites obtained from the non-cooperative McGhee-von Hippel analysis of the spectrophotometric data (Table 1).

### Optical Thermal Denaturation Studies and Analysis of the Stabilization Effects

**Figure 4:**
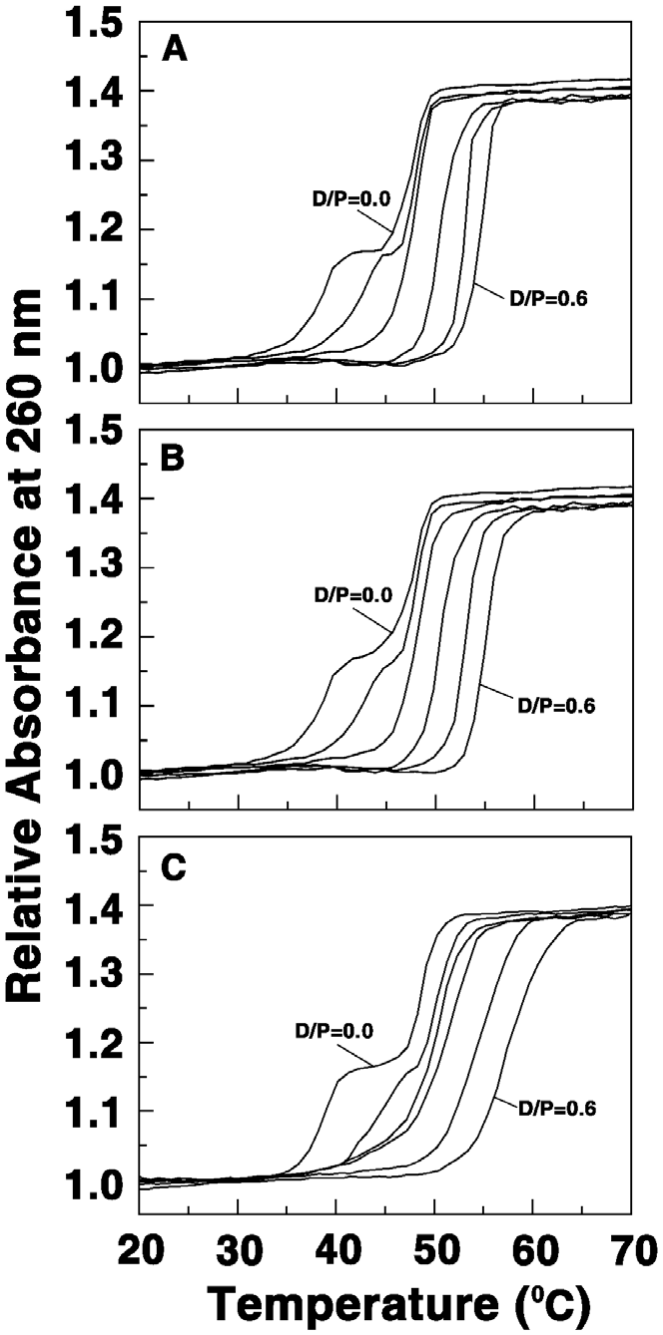
Thermal melting profiles of triplex with BC and BC analogs. Poly(U)_•_poly(A)_*_poly(U) (30.0 μM) treated with (A) BC (B) BC1 and (C) BC2. Curves 1–6 represent D/P in the range 0–0.6.

A thermal denaturation experiment can determine the stability of the triplex and duplex structures. *T*_m_ is defined as temperature of melting or, more accurately, as temperature of mid-transition. Beyond a simple numerical value (the *T*_m_), a thermal denaturation experiment, that depicts the variation of the absorbance plotted vs. temperature, yields important thermodynamic information. The third strand [poly(U)] separation from the triplex occurs at 35°C while the duplex strand denaturation occurs at 45°C under the present experimental conditions and this is in agreement with the literature data [32]. The stability of triplex in presence of BC and the analogs was monitored by UV thermal melting curves that are presented in Figure 4. It can be seen that the analogs effected remarkable stabilization of the third strand. The extent of stabilization was high for BC1 and higher for B2 compared to BC suggesting a delayed strand separation with bound analogs. The stabilization temperature for the third strand in presence of BC, BC1 and BC2 were 17.5, 18.1 and 20.5°C, respectively. Detailed melting data is presented in Table 2.

**Table 2:** Effect of alkaloids on the thermal stability of the poly(U)_•_poly(A)_*_poly(U) triplex^a^.

Complex	D/P	*T_m_*’/(°C) 3 → 2	*T_m_*’’/(°C) 2 → 1	Δ*T_m_*’/(°C) 3 → 2	Δ*T_m_*’’/(°C) 2 → 1
Triplex alone	0	37.0	47.5	–	–
Triplex + BC	0.1	42.0	48.0	5.0	0.5
	0.2	46.8	–	9.7	–
	0.4	50.1	–	13.1	–
	0.5	52.9	–	15.9	–
	0.6	54.5	–	17.5	–
Triplex +BC1	0.1	42.2	48.3	5.2	0.8
	0.2	48.0	–	11.0	–
	0.4	50.6	–	13.6	–
	0.5	53.1	–	16.1	–
	0.6	55.1	–	18.1	–
Triplex + BC2	0.1	44.0	50.0	7.0	2.5
	0.2	49.6	–	12.6	–
	0.4	41.0	–	14.0	–
	0.5	54.3	–	17.3	–
	0.6	57.5	–	20.5	–

a Average from three experiments. Error limits for individual *T_m_* measurements are estimated to be within ±0.5°C in. *T_m_’* (3 → 2) and *T_m_”* (2→1) are triplex to duplex and duplex to single stranded transitions respectively. Δ*T_m_*  =  (*T_m_* of triplex-alkaloid complex – *T_m_* of triplex or duplex).

### Binding Mode of Berberine Analogs from Fluorescence and Viscosity Studies

**Figure 5:**
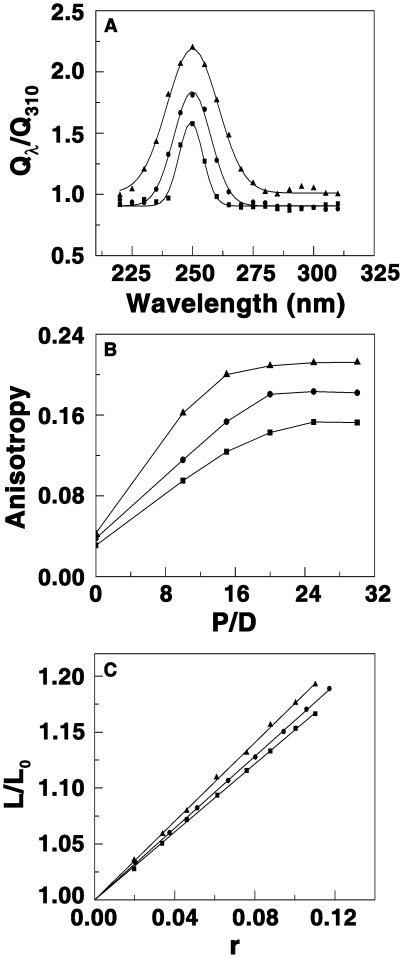
Variation of the relative fluorescence quantum yields, anisotropy and visocisty. (A) Variation of the relative fluorescence quantum yield of BC (▪), BC1 (•), and BC2 (▴) in the presence of poly(U)_•_ **.**poly(A)_*_poly(U) as a function of excitation wavelength. (B) A plot of the variation of anisotropy values versus P/D ratio for the complexation of BC (▪), BC1 (•), BC2 (▴) with poly(U)_•_poly(A)_*_poly(U). (C) A plot of increase in helix contour length (L/Lo) versus r for the complexation of BC (▪), BC1 (•), BC2 (▴) with poly(U)_•_poly(A)_*_poly(U) at 25±0.5°C.

Although the above experiments indirectly suggest intercalation model of interaction, additional experiments aimed at the elucidation of intercalation mode were performed by fluorescence energy transfer, quenching, and viscosity studies. Energy transfer from the base triplets to the bound alkaloid analogs can be used as an evidence for intercalative binding. Efficient energy transfer manifested by an increased quantum yield can occur only when the bound alkaloid molecules are oriented parallel to the base triplets in an intercalated manner. In Figure 5A the variation of Q_λ_/Q_310_ with wavelength for the complexation of BC and analogs at a D/P (alkaloid/RNA triplex molar ratio) of 1.0 is presented. An increase in the quantum yield is revealed in the 230–280 nm region with maximum around 258 nm which is the RNA absorbing region. The increase in quantum yield was higher for BC1 and the highest for BC2 compared to BC indicating that the binding of these molecules results in significant energy transfer to the base triplets providing clear evidence for a better intercalated orientation of the analogs compared to BC.

Further support for stronger intercalation of the analogs was gathered from fluorescence polarization anisotropy experiments. The polarization of the complexes enhanced as the D/P increased (Figure 5B) and the values were 0.15, 0.18 and 0.21 confirming the intercalated binding and the higher binding of BC over BC1 and BC2.

The mode of binding was subsequently confirmed to be intercalative from viscosity studies also. Intercalation of molecules between DNA bases is known to increase the viscosity of the solution owing to unwinding and elongation of the double helix as proposed by Lerman [77]. This is due to the fact that intercalation leads to enhancement of the axial length of the nucleic acid resulting in more rigidity due to enhancement of the frictional coefficient. The binding mode of the BC analogs to the RNA triplex was probed by measuring the viscosity of the triplex solution in the presence of increasing concentrations of the alkaloids, and estimating the changes in relative viscosities with varying D/P ratio. The change was found to be more rapidly pronounced (not shown) for the analogs compared to berberine complexes, and the saturation was achieved at a much lower D/P ratio indicating tighter intercalative binding of the analogs. It is evident from Figure 5C that the binding of the analogs results in an increase in the relative viscosity of the solution. Viscosity results are expressed as length enhancements with respect to a standard value (β) of 2.0 for a true intercalator resulting in a theoretical length enhancement of 0.34 nm. The β values for BC1 and BC2 triplex complexes were 1.60, and 1.76, respectively, against a value of 1.51 for BC. Thus, a better intercalation model may be envisaged for BC1 and 2 binding to the triplex, whereas weak intercalation may be assigned to the binding of BC. Although BC and analogs have partial saturation in the ring structure resulting in buckled structure a better intercalation appears to be feasible with the analogs contributed by the anchoring of the side chain and the above described experiments unequivocally confirm a better intercalation geometry for the analogs compared to BC.

Additionally, fluorescence quenching experiments [78] which is also a reliable method for studying the binding mode of small molecules to nucleic acids were performed to lend support to the intercalation mode. Generally, the molecules bound to the surface of the triplex by electrostatic interaction will be easily accessible to a quencher while those bound inside the helix will be protected from the quencher [78], [79]. It is known that anionic quenchers like [Fe(CN)_6_]^4−^ cannot access the inside of the helix due to the strong electrostatic repulsion from the negatively charged phosphate groups and consequently very little quenching will be observed for fluorescent small molecules bound in the interior of the triplex. Consequently, the magnitude of the Stern-Volmer quenching constant (*K*_sv_) of ligands that are bound inside by intercalation will be lower than that of the free molecules. In Figure S2, the data on the fluorescence quenching of the berberine analogs in presence of K_4_[Fe(CN)_6_] is presented. *K*_sv_ values of free BC1 and BC2 with [Fe(CN)_6_]^4−^ were 217 M^−1^, respectively, while that of bound ones were 73.0 and 42.0 M^−1^, respectively, indicating that the bound berberine analogs are considerably protected and sequestered away from the solvent. This result further suggests an intercalative or similar kind of binding for the analogs inside the triplex.

### Determination of Quantum Efficiency

The quantum efficiency (Q) ligand of a binding to nucleic acid is a measure of the energy transferred from to the ligand upon complexation. This is obtained from the ratio of the quantum efficiency of ligand complexed to nucleic acid (q_b_) to the quantum efficiency of the free ligand (q_f_). Quantum efficiency values are important information that may support the strong binding of the analogs to the RNA triplex. A plot of ΔA against the inverse of RNA triplex concentration gave an exponential plot (not shown) from which quantum efficiency values of 60.0, 44.9 and 22.8, respectively, for BC, BC1, and BC2 have been determined. Since Q is >1.0 this indicates enhancement of fluorescence intensity and greater retention of fluorescence energy by the bound alkaloid due to shielding within the binding site from quenching by solvent.

### Conformational Aspects of the Binding

**Figure 6:**
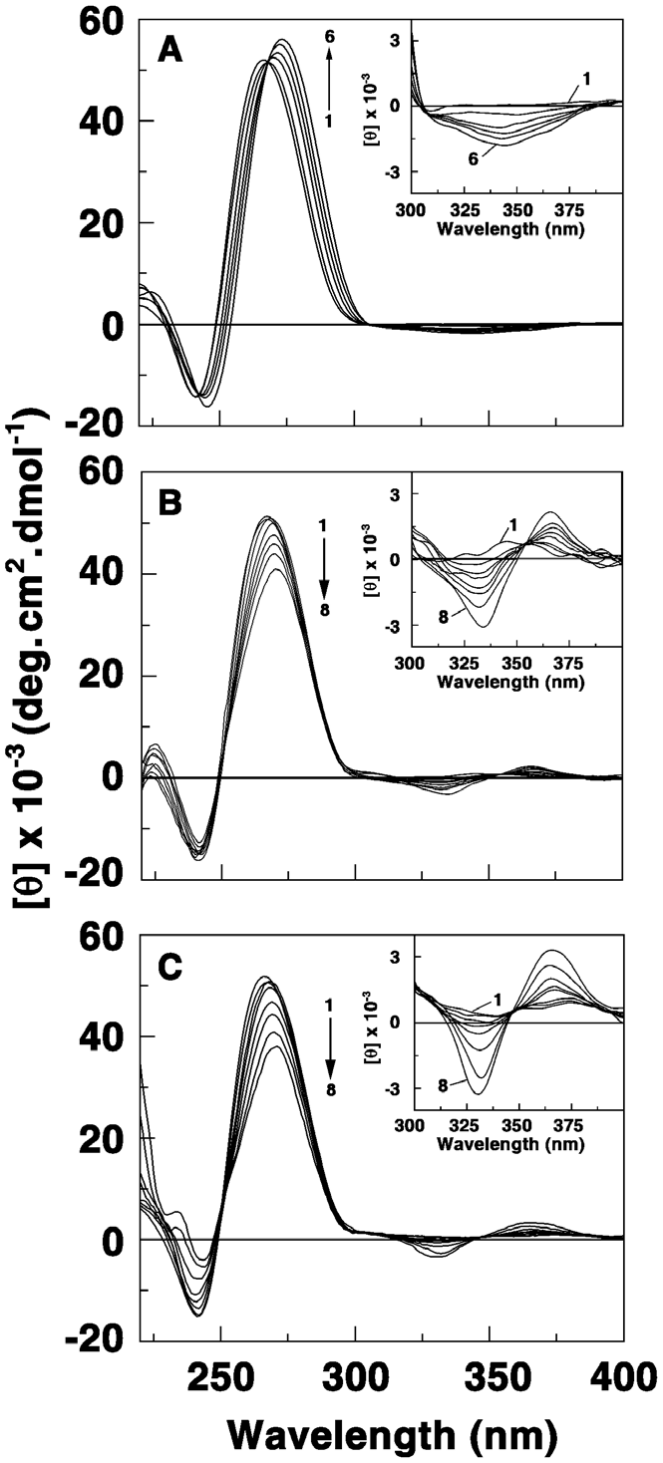
Circular dichroic spectral titration of triplex with BC and BC analogs. Titration data of poly(U)_•_poly(A)_*_poly(U) (30 µM) treated with (A) 0.0, 3.0, 6.0, 9.0, 12.0, 18.0 µM of BC (B) 0.0, 3.0, 6.0, 9.0, 12.0, 15.0, 18.0, 24.0 µM of BC1 and (C) 0.0, 3.0, 6.0, 9.0, 12.0, 15.0, 18.0, 24.0 µM of BC2. Inset: A magnified representation of the induced CD spectra in the region 300–400 nm.

Conformational changes associated with the binding of these analogs in comparison to berberine were studied from circular dichroism studies. The circular dichroic spectrum of the triplex was characterized by a positive band around 270 nm followed by a small negative band around 240 nm. (Curve 1, Figure 6A). The spectrum is in conformity with the literature report. In the presence of BC an enhancement and red shift of the positive band and a small change in the ellipticity of the negative band occurs. A small induced CD band is developed in the 320–360 nm with a maximum around 345 nm. In the presence of BC1, the positive ellipticity of the triplex decreased with a concomitant red shift of the maximum (Figure 6B). There was a small decrease of the negative band maximum around 240 nm. The overall changes in the CD spectrum were more pronounced compared to the changes with BC. An induced CD band appeared in the 320–360 nm, but was of an excision split type with a negative and positive ellipticity around 340 and 365 nms. For the binding of BC2, the intrinsic CD band ellipticity of the triplex around 270 nm decreased significantly with a concomitant red shift of the peak maximum (Figure 6C). The 240 nm band ellipticity also reduced in intensity. The appearance of the induced CD bands was similar to that with BC1, but the intensity was more pronounced. From the comparative CD curves it is clear that the binding induced CD perturbation of the triplex structure was highest with BC2 and followed the order BC2>BC1>BC. Furthermore, the pronounced induced CD band and the differences in their appearance indicate the analogs bind strongly to the triplex and are in line with the 15 times higher binding affinity of the latter. It also appears that the bound orientation of BC analogs on the triplex structure is significantly different from that of BC as revealed by different induced CD patterns that reflects the interaction of the transition moments of the bound molecule and the base triplets.

**Figure 7:**
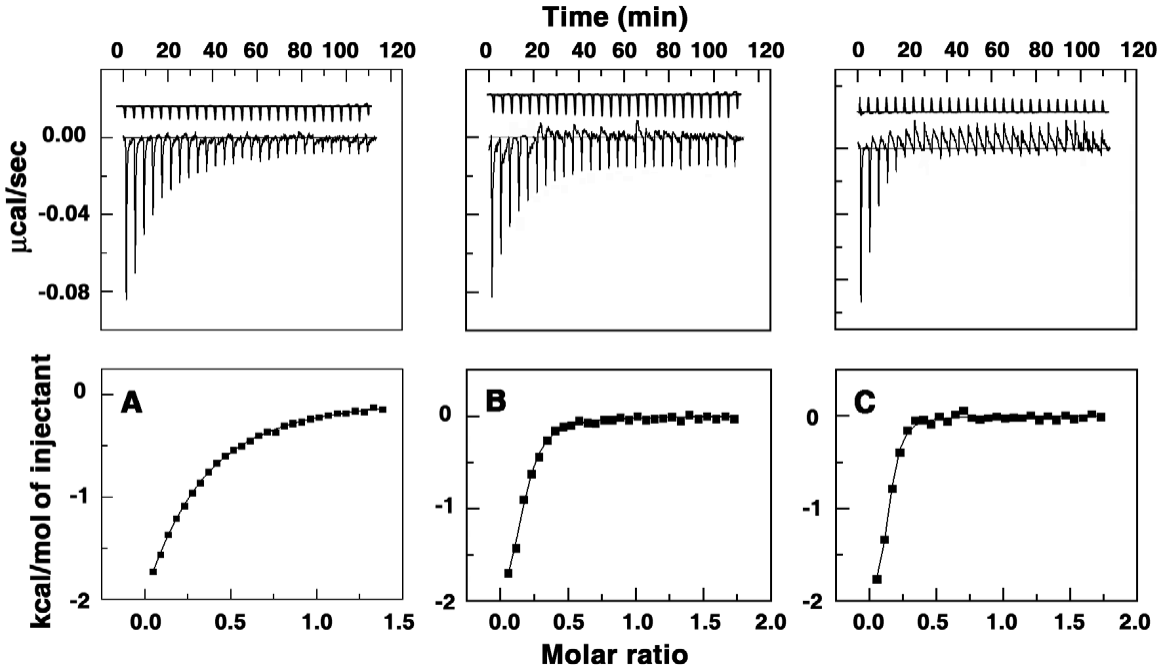
Representative isothermal titration calorimetry profiles for the titration of BC and analogs to triplex. Titration profiles are shown for poly(U)_•_poly(A)_*_poly(U) with (A) BC (B) BC1 and (C) BC2 at 25°C. The top panels represent raw data for the sequential injection of the alkaloid into the triplex solution. The lower panels represent the corresponding normalized heat signals versus molar ratio. The data points (▪) reflect the experimental injection heat while the solid lines represent the calculated fit of the data.

**Table 3:** ITC Derived Thermodynamic parameters for the binding of BC, BC1 and BC2 with poly(U)_•_poly(A)_*_poly(U) triplex at 25°C.^a^

Parameters	BC	BC1	BC2
***K_a_ *** **/10^5 ^m** ^−**1**^	1.5±0.04	9.73±0.09	23±0.10
**N**	4.7	4.5	4.5
**Δ** ***G*** **^o^ (kcal mol** ^−**1**^ **)**	−7.10±0.02	−8.22±0.04	−8.73±0.05
**Δ** ***H*** **^o^ (kcal mol** ^−**1**^ **)**	−4.86±0.03	−2.30±0.05	−1.65±0.05
***T*** **Δ** ***S*** **^o^ (kcal mol** ^−**1**^ **)**	2.24	5.92	7.08

a All the data in this table are derived from ITC experiments and are average of four determinations. *K_a_* and Δ*H* ^o^ values were determined from ITC profiles fitting to Origin 7 software as described in the text. n is the site size which is the reciprocal of the stoichiometry N. The values of Δ*G* ^o^ and TΔ*S* ^o^ were determined using the equations Δ*G* ^o^  =  − RTln *K_a_* and Δ*G* ^o^ *= *Δ*H* ^o^ − TΔ*S* ^o^. All the ITC profiles were fit to a model of single binding site.

### Binding Thermodynamics: Isothermal Titration Calorimetry

Thermodynamic characterization of the binding was performed from isothermal titration calorimetry. The calorimetric profiles for the binding of BC1 and BC2 to the RNA triplex are presented in Figure 7. Binding in each case was characterized by the presence of exothermic peaks that followed each injection of the alkaloid to the triplex solution. Fits of the integrated heat data were carried out using a model of single set of identical sites that yielded a fairly good fitting of the experimental data. The thermodynamic parameters elucidated for the binding of the alkaloids to the triplex are collated in Table 3. The ITC data for the binding of BC to the triplex yielded an association constant of (1.50±0.04×10^5^ m ^−1^). This is in close agreement to the value reported previously [32]. The binding of BC1 yielded a six times higher association constant of (9.73±0.09×10^5^ m ^−1^) while the binding of BC2 was remarkably stronger compared to BC and BC1 with a *K_a_* value of (2.30±0.1×10^6^ m ^−1^) showing almost fifteen times stronger binding than BC. The affinity values are in excellent agreement with the values from spectroscopic results. The number of binding sites obtained from ITC as reciprocal of stoichiometry were closely comparable to the number of excluded sites from spectroscopic data and also the stoichiometry data from Job plot. The Gibbs energy change (Δ*G* ^o^) was found to ∼−7.1 kcal/mol for BC but higher at −8.22 kcal/mol for BC1 and 8.73 kcal/mol for BC2 showing the spontaneity of the interaction in all cases in general and more for BC1 and BC2. The binding of BC to triplex was predominantly enthalpy driven with a small favorable entropy change as reported previously [32]. On the other hand, binding of BC1 to the triplex was driven by a large positive entropy change (5.92 kcal/mol) and a small enthalpy change. It may be recalled that CD spectroscopy had indicated remarkable conformational changes of triplex on binding of BC1 and BC2 compared to BC. The overall entropy change may be expected from this conformational change. Binding of BC1 and BC2 has been revealed to result in stronger intercalation complex compared to BC and consequently a larger lengthening, stiffening, and rigidifying of the triplex may be the most likely explanation for the entropic cost as suggested by Chaires [80]. This was also confirmed from our viscosity studies where the length enhancement of the triplex due to unwinding with BC1 and BC2 was much higher than with BC.

In conclusion, we report here a remarkably higher binding affinity of two new 9-substituted berberine analogs to the RNA triplex of poly(U)**.**poly(A)_*_poly(U). This has also been complemented by higher melting stabilization of the third stand, stronger conformational changes of the triplex structure and generation of stronger induced CD bands for the triplex bound analogs. The overall higher affinity and stabilization by the analogs were clearly reflected in the thermodynamic parameters of the complexation elucidated from calorimetry. Results suggest that the length of the alkyl side chain clearly influence in the triplex stabilization phenomena of BC analogs. These results may be useful for formulating effective antigene strategies involving new berberine derivatives and the RNA triplex.

## Supporting Information

Figure S1 **Job plot for the complexation of BC, BC1 and BC2 with the RNA triplex.**
(TIF)

Figure S2 **Stern-Volmer plots for the quenching of BC, BC1 and BC2 by RNA triplex.**
(TIF)

Table S1 **Optical properties of free and triplex bound alkaloid analogs.**
(DOC)
