# Supplementary figures and images for: Biophysical Characterization of the Strong Stabilization of the RNA Triplex poly(U)•poly(A)*poly(U) by 9-O-(ω-amino) Alkyl Ether Berberine Analogs
Source: PLoS One. 2012 May 29;7(5):e37939. doi: 10.1371/journal.pone.0037939 (PMC3362543; doi:10.1371/journal.pone.0037939)

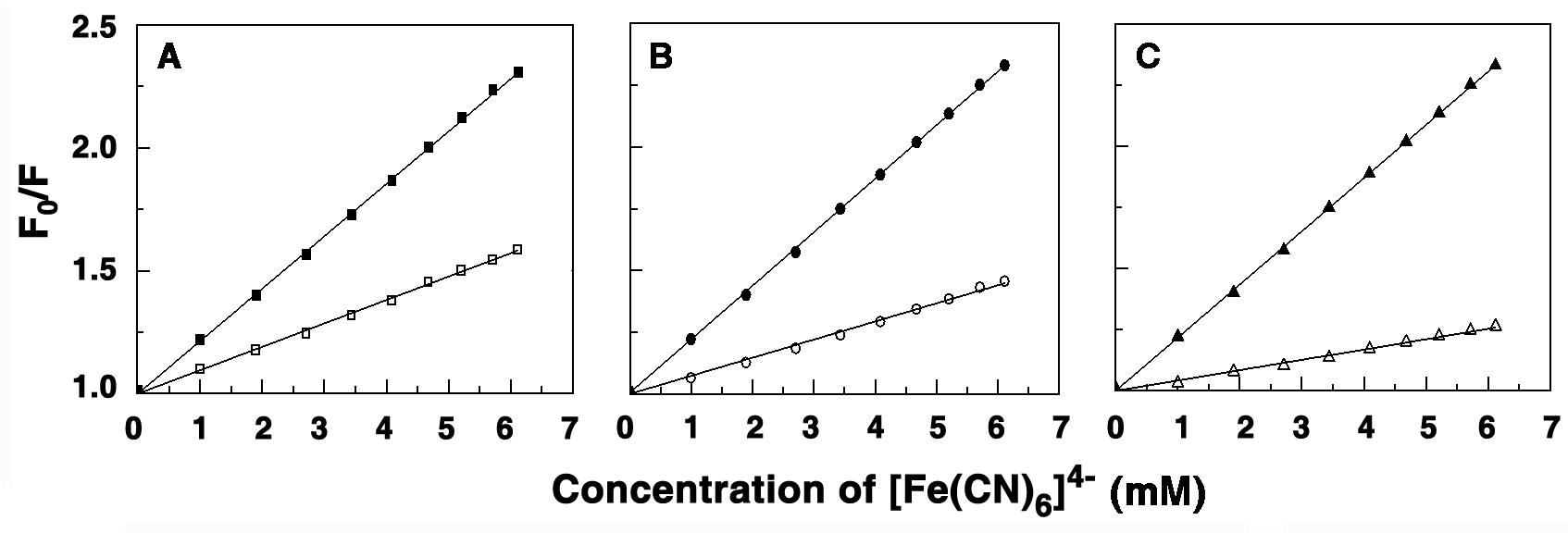

Supplement: Figure S2 — Stern-Volmer plots for the quenching of BC, BC1 and BC2 by RNA triplex. (TIF) [file pone.0037939.s002.tif]
