# Supplementary material for: Biophysical Characterization of the Strong Stabilization of the RNA Triplex poly(U)•poly(A)*poly(U) by 9-O-(ω-amino) Alkyl Ether Berberine Analogs
Source: PLoS One. 2012 May 29;7(5):e37939. doi: 10.1371/journal.pone.0037939 (PMC3362543; doi:10.1371/journal.pone.0037939)
Supplement: Table S1 — Optical properties of free and triplex bound alkaloid analogs. (DOC) [file pone.0037939.s003.doc]

Table S1: Summary of the optical properties of free and Poly(U).Poly(A)*Poly(U) bound alkaloids.

| Parameter^a^ | BC | BC1 | BC2 |
| --- | --- | --- | --- |
| **Absorbance** | | | |
| λ_max_(free) | 345 | 345 | 345 |
| λ_max_(bound) | 349 | 350 | 350 |
| λ_iso_^b^ | 356,381,440 | 357,377,454 | 354,380,436 |
| ε_f_ (at λ_max_) | 22,500 | 22,500 | 22,500 |
| ε_b_(at λ_max_) | 14,512 | 15,615 | 15,040 |
| **Fluorescence** | | | |
| λ_max_(excitation) | 444 | 447 | 446 |
| λ_max_(emission) | 513 | 516 | 515 |

^a^Units: λ, nm; ε(molar extinction coefficient), M^-1^cm^-1^. ^b^Wavelengths at the isosbestic point.
